# Gelatinous pseudocysts in cryptococcal meningoencephalitis

**DOI:** 10.1590/0037-8682-0201-2020

**Published:** 2020-11-13

**Authors:** Bruno Niemeyer de Freitas Ribeiro, Edson Marchiori

**Affiliations:** 1 Instituto Estadual do Cérebro Paulo Niemeyer, Departamento de Radiologia, Rio de Janeiro, RJ, Brasil.; 2 Grupo Fleury, São Paulo, SP, Brasil.; 3 Universidade Federal do Rio de Janeiro, Departamento de Radiologia, Rio de Janeiro, RJ, Brasil.

A 26-year-old man was admitted with fever and headache that persisted for 2 weeks, progressing with reduced consciousness and seizures. Serological tests for HIV showed positive results and the CD4 count was 76 cells/mm^3^. The serological test for syphilis showed negative results. Cerebrospinal fluid analysis revealed pleocytosis (42/mm³) with lymphocytic predominance, increased protein concentration (125 mg/mL), a negative VDRL test result, and a positive cryptococcal capsular polysaccharide antigen test result with fungal culture positive for *Cryptococcus neoformans.* Brain magnetic resonance imaging showed multiple intra-axial lesions scattered throughout the cerebral hemispheres (Figure A and Figure B) without significant enhancement with gadolinium (Figure C), suggesting perivascular impairment due to gelatinous pseudocysts. Treatment with intravenous amphotericin B deoxycholate 0.7 mg/kg/d and flucytosine 100 mg/kg/d was initiated, but the patient died 1 week later.

**FIGURE 1:**
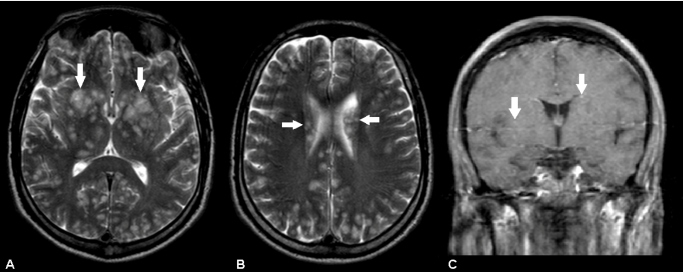
Multiple intra-axial lesions scattered throughout the cerebral hemispheres, sometimes confluent, characterized by a hyperintense T2 signal **(A and B)** without significant enhancement with gadolinium **(C)**. Such lesions have no significant mass effect and perilesional edema, suggesting perivascular impairment due to gelatinous pseudocysts. Absence of meningeal enhancement (**C**), a common finding in cryptococcal meningoencephalitis in immunocompromised patients.

Cryptococcosis is a disease caused by *Cryptococcus neoformans*, an encapsulated yeast that usually affects immunocompromised patients. It is the third most common intracranial pathogen in acquired immunodeficiency syndrome (AIDS) patients, only surpassed by HIV itself and *Toxoplasma gondii* ^1^ ^-^ ^3^. In AIDS patients, cryptococcal infection generally manifests as meningoencephalitis or a disseminated disease. Meningeal infection may involve the brain parenchyma or may extend along the Virchow-Robin spaces, causing dilation of perivascular spaces due to mucous gelatinous material produced by the fungal capsule. In these cases, neuroimaging studies show multiple, small, round/oval lesions in the basal ganglia and thalami nucleus without significant enhancement with gadolinium^1^ ^-^ ^3^.

Cryptococcosis should be considered in the differential diagnosis in immunocompromised patients with dilated Virchow-Robin spaces.
